# Deep-learning-based automatic segmentation and classification for craniopharyngiomas

**DOI:** 10.3389/fonc.2023.1048841

**Published:** 2023-05-05

**Authors:** Xiaorong Yan, Bingquan Lin, Jun Fu, Shuo Li, He Wang, Wenjian Fan, Yanghua Fan, Ming Feng, Renzhi Wang, Jun Fan, Songtao Qi, Changzhen Jiang

**Affiliations:** ^1^ Department of Neurosurgery, First affiliated Hospital of Fujian Medical University, Fuzhou, Fujian, China; ^2^ Department of Medical Image Center, Southern Medical University, Nanfang Hospital, Guangzhou, China; ^3^ Department of Plastic Surgery, Peking Union Medical College Hospital, Beijing, China; ^4^ Department of Neurosurgery, Peking Union Medical College Hospital, Beijing, China; ^5^ Department of Neurosurgery, Xuanwu Hospital, Capital Medical University, China International Neuroscience Institute, Beijing, China; ^6^ Department of Neurosurgery, Beijing Tiantan Hospital, Beijing Neurosurgical Institute, Capital Medical University, Beijing, China; ^7^ Department of Neurosurgery, Southern Medical University, Nanfang Hospital, Fuzhou, Fujian, China

**Keywords:** craniopharyngiomas, QST typing system, deep learning, segmentation, classification

## Abstract

**Objective:**

Neuronavigation and classification of craniopharyngiomas can guide surgical approaches and prognostic information. The QST classification has been developed according to the origin of craniopharyngiomas; however, accurate preoperative automatic segmentation and the QST classification remain challenging. This study aimed to establish a method to automatically segment multiple structures in MRIs, detect craniopharyngiomas, and design a deep learning model and a diagnostic scale for automatic QST preoperative classification.

**Methods:**

We trained a deep learning network based on sagittal MRI to automatically segment six tissues, including tumors, pituitary gland, sphenoid sinus, brain, superior saddle cistern, and lateral ventricle. A deep learning model with multiple inputs was designed to perform preoperative QST classification. A scale was constructed by screening the images.

**Results:**

The results were calculated based on the fivefold cross-validation method. A total of 133 patients with craniopharyngioma were included, of whom 29 (21.8%) were diagnosed with type Q, 22 (16.5%) with type S and 82 (61.7%) with type T. The automatic segmentation model achieved a tumor segmentation Dice coefficient of 0.951 and a mean tissue segmentation Dice coefficient of 0.8668 for all classes. The automatic classification model and clinical scale achieved accuracies of 0.9098 and 0.8647, respectively, in predicting the QST classification.

**Conclusions:**

The automatic segmentation model can perform accurate multi-structure segmentation based on MRI, which is conducive to clearing tumor location and initiating intraoperative neuronavigation. The proposed automatic classification model and clinical scale based on automatic segmentation results achieve high accuracy in the QST classification, which is conducive to developing surgical plans and predicting patient prognosis.

## Highlights

• A segmentation method was used to segment six tissues with a mean Dice value of 0.8668.

• A classification model was used to predict the QST subtypes with an accuracy of nasa 0.9098.

• A clinical scale to predict the QST subtypes with an accuracy of 0.8647.

## 1 Introduction

Craniopharyngiomas (CPs) arise from tumors of the epithelial cells of Rathke’s capsule and account for 2%–5% of primary intraluminal tumors (1, 2). Despite being pathologically benign, these tumors can be aggressive and locally affect important structures, including the hypothalamic–pituitary axis, and cause serious postoperative complications (3–5). The standardized mortality ratio (SMR) decreased significantly after 2010, but an SMR of 2.9 and serious complications still cannot be ignored (6).

At present, many studies classify CPs according to the impact characteristics, but according to our research, only a small number of current studies have compared the impact of different surgical methods on different types of CPs, including the transcranial approach (TCA) and the endoscopic endonasal approach (EEA) (7–9). A recent study classified CPs into three types according to the QST classification system based on the origin of the tumor: 1) infrasellar/subdiaphragmatic CPs (Q-CPs), which arise from the subdiaphragmatic infrasellar space with an enlarged pituitary fossa; 2) subarachnoidal CPs (S-CPs), which arise from the middle or inferior segment of the stalk and tend to extend among cisterns; and 3) pars tuberalis CPs (T-CPs), which arise in the top of the pars tuberalis, mainly extend upward, and occupy the third ventricular compartment (10, 11).

The OST classification system advances our knowledge of the morphological traits, growth patterns, and actual connections between CPs and the hypothalamic–pituitary axis. Based on the QST classification, the researchers found a relationship with prognosis. They found that EEA in the Q-CPs increased the rate of tumor resection and had a greater probability of visual improvement, while TCA was recommended in the T-CPs with a better prognosis for hypothalamic function (12–14). T-CPs were also reported with sodium disturbance (15). Therefore, an accurate diagnosis of the QST classification before surgery will be important for surgeons in selecting the surgical approach to maximize patients’ quality of life after surgery.

However, the preoperative identification of tumor types requires accumulating clinical experience in large series, which is an unavoidable limitation for most institutions. Furthermore, many CP cases with different QST types have similar morphology, behave similarly on conventional preoperative examination, and bring difficulties for even experienced hands. Therefore, using new image-based methods to accurately classify QST types of tumors preoperatively is of great value to clinicians.

Currently, methods based on radiomics and deep learning (or machine learning) are increasingly used in neurosurgery and show promising clinical application value, such as neuronavigation and prognostic analysis (16–18). Previous studies have performed structural segmentation for CPs or pathological classification (19, 20). However, there is still a lack of a multi-structure segmentation method for CPs and a deep learning model based on the QST classification, which significantly limits visual analysis and surgical decision-making.

## 2 Materials and methods

**Figure 1:**
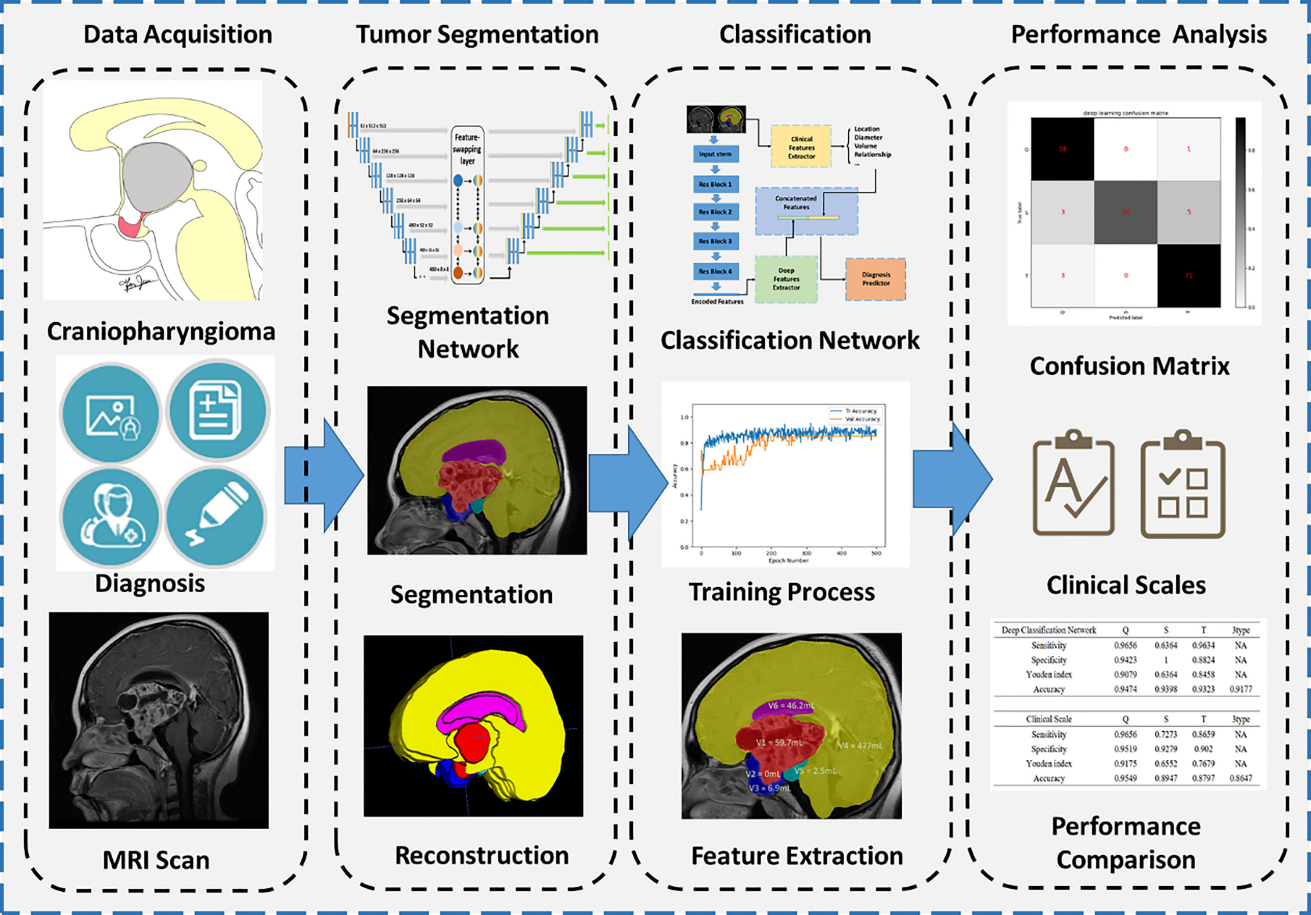
Flowchart of segmentation and classification. Four main steps are illustrated, including data acquisition, tumor segmentation, classification, and performance analysis.

An overall flowchart was built and illustrated in **Figure 1**, including data acquisition, tumor segmentation, classification, and performance analysis.

### 2.1 Participants

A total of 133 patients diagnosed with craniopharyngioma at the Affiliated Hospital of Southern Medical University were enrolled in this study. Surgeons designed different surgical plans according to the size and location of the tumor, including transsphenoidal surgery or craniotomy. According to the specific criteria of the QST classification, the surgeons carefully evaluated the origin of the tumor for each patient during surgery. The basic clinical characteristics of patients were also collected in this study. The study was reviewed and approved by the Ethics Committee of the Affiliated Hospital of Southern Medical University.

### 2.2 Segmentation protocol of MRI

According to clinical experience, preoperative classifications are established based on the sagittal view of MRI. In this study, we used sagittal MRI scans as the main imaging data. All patients were scanned by a 3.0-T scanner with a pixel size of 0.45 mm and a slice thickness of 6 mm. To accurately localize and classify the tumor, we manually labeled the T1-enhanced MRI images into seven classes, including background:

0: Background, i.e., non-labeled component.
1: Tumor: containing all tumor components, such as enhanced cyst walls and heterogeneous cysts.
2: Pituitary: considering that the pituitary stalk is relatively small and difficult to label alone; the pituitary stalk and pituitary gland were classified into the same category.
3: Sphenoid sinus, including the air space of the sphenoid sinus and the bony structure (the dura surrounding the pituitary fossa).
4: Brain: the normal brain structure, including the cerebrum, cerebellum, and brainstem,
5: Suprasellar cistern: a structure around the pituitary stalk, above the pituitary gland, and below the brain, containing the suprasellar cistern and the interpedal cistern. This structure can be absent due to tumor compression.
6: Ventricle: lateral ventricles.

The annotations were made by three physicians with 7 years of neuroimaging experience and verified by one physician with 20 years of neuroimaging experience.

### 2.3 Establishment of the automatic segmentation model

**Figure 2:**
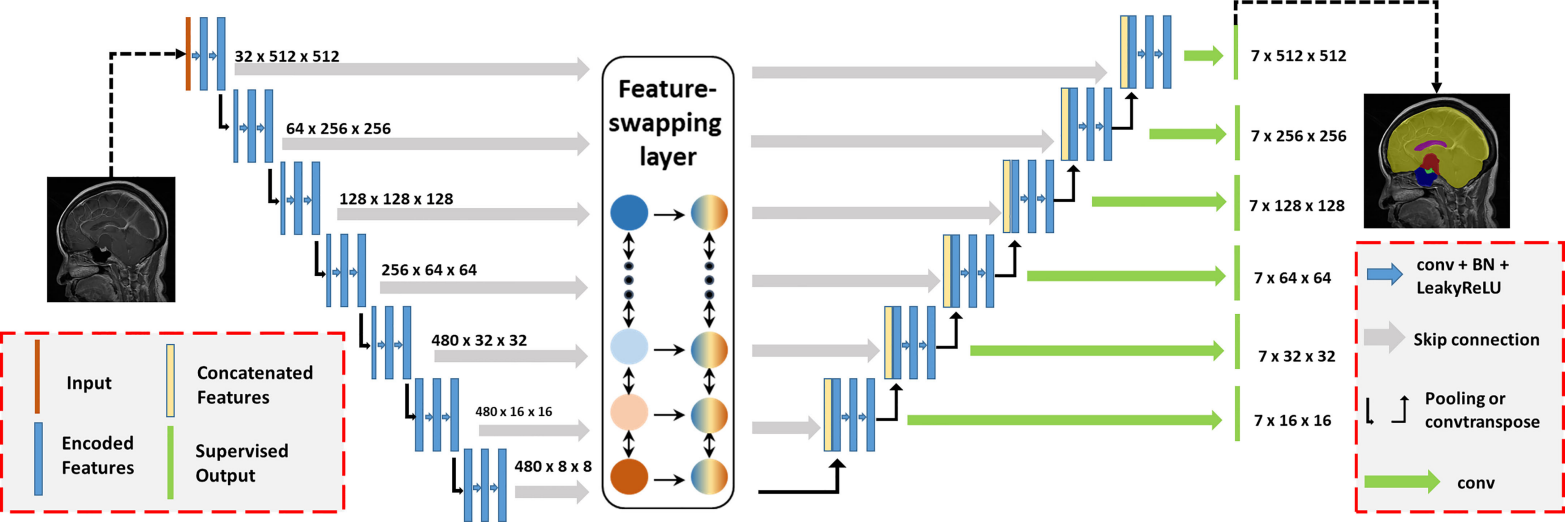
Automatic segmentation network structure for craniopharyngiomas. The proposed model was based on the nnUNet backbone with deep supervision. We proposed a feature-swapping layer to exchange the features of different resolutions. First, the input (MR image) was encoded to features with different resolutions. After swapping features, the encoded features were decoded to the segmentation map as the final output.


Since the sagittal MRI has a large slice thickness, we transformed the entire 3D MRI into several 2D slices for training. However, the volumes of different tissues varied among CPs; for example, the volume of the brain was thousands of times larger than the volume of the pituitary. Excessive downsampling layers in convolutional neural networks (CNNs) would lose positional information on small volumes of tissues caused by such class imbalance. Therefore, we adopted nnUNet as the backbone CNN and proposed a feature-swapping layer to exchange the features of different resolutions extracted in the encoder (**Figure 2**) (21). The exchanged features can effectively integrate different levels of semantic information for accurate segmentation of structures with small volumes. The original MRI was input to training with fivefold cross-validation, deep supervision was used to increase the stability of the training, and the Dice coefficient was used as the judgment criterion for the separation accuracy.

$$Dice\;coefficient=\;\frac{2\left|A\cap^{}B\right|}{\left|A\right|+\left|B\right|}$$

where A and B represent the manual segmentation and deep learning results, respectively. The training process for each fold took approximately 15 h on an NVIDIA 3090 GPU with 24 GB of random-access memory.

### 2.4 Feature extraction

Based on the results of automatic segmentation augmented with minor modifications by experts, we performed feature extraction for each patient, including the volume, location, and diameter of six segmented tissues. In particular, according to the growth characteristics of tumors classified by the QST classification, we proposed several characteristics based on experts’ clinical views that may be of important significance: the volume of tumors anterior to the tuberculum sellae, the volume of tumors occupying the pituitary fossa, the morphology of tumors (referring to regular pyramidal structure or inverted pyramid structure, we used the relative location of the maximum transverse diameter of the tumor to describe this feature), the aspect ratio of tumors, the location of tumors relative to the brain, and the location of tumors relative to the sellar region.

### 2.5 Establishment of the classification model

**Figure 3:**
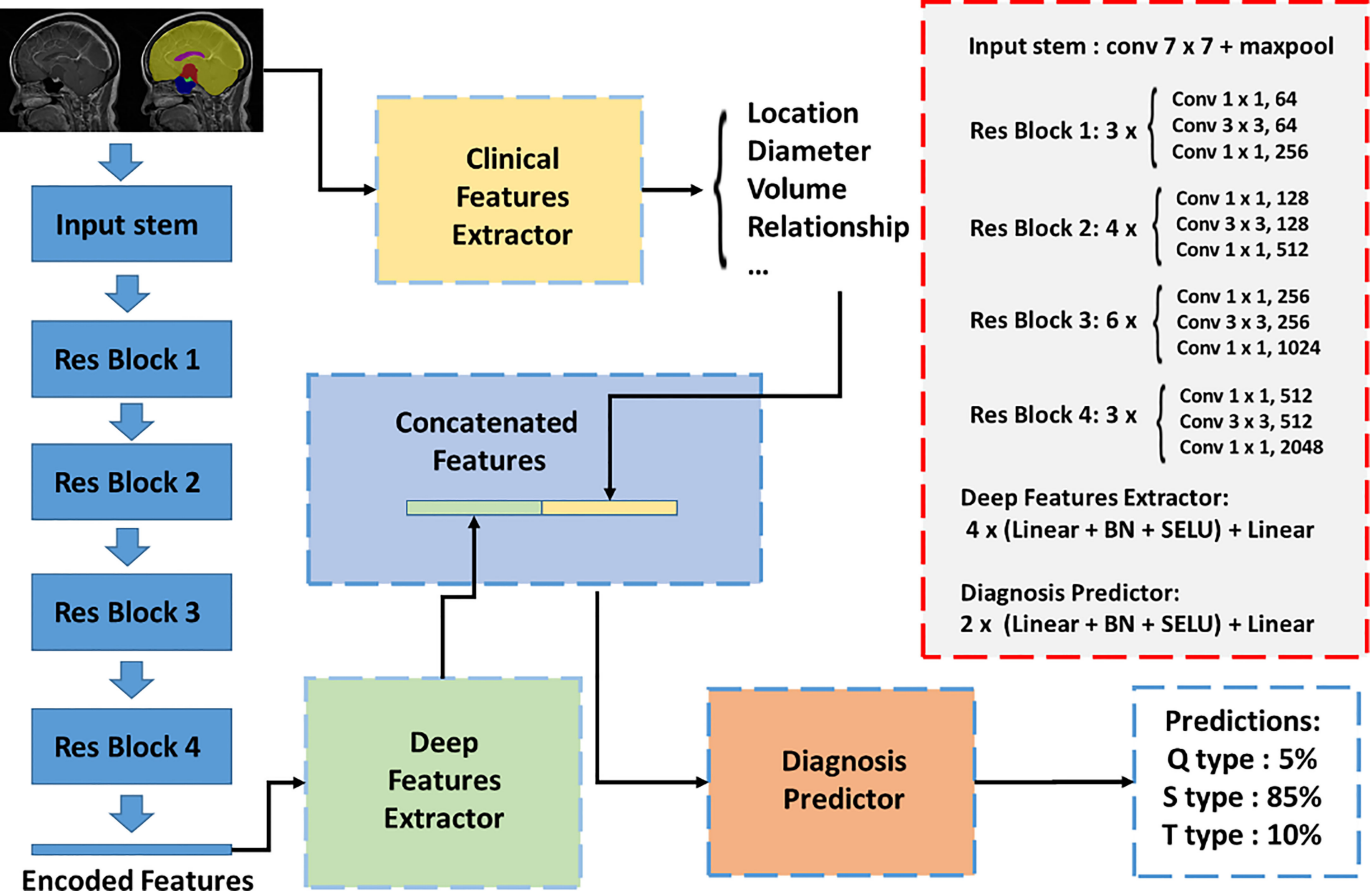
Automatic classification network structure for QST classification. The inputs of the classification network contained raw MRI data, autosegmented images with manual modification, and automatically extracted clinical knowledge-based features. An image feature extraction module was proposed based on the Resnet50 backbone. Extracted features from the image feature extraction module and clinical knowledge-based features were fed into the final discriminator for classification, and the probability of each type was output.

In this study, we presented a classification network to integrate multimodal inputs (**Figure 3**). The inputs of the classification network contained raw MRI data, autosegmented images with manual modifications, and automatically extracted clinical knowledge-based features. Since features with different modalities cannot be concatenated directly, we first set up an image feature extraction module based on the ResNet50 backbone. The sagittal slice with the largest tumor area of each patient was selected. Segmentation results of sagittal slices were fed into the classification network after one-hot coding. Extracted features from the image feature extraction module and clinical knowledge-based features were fed into the final discriminator for classification, and the probability of each type of class was output. We adopted the three-class CrossEntropy loss as the loss function. The classification network also applied fivefold cross-validation for training, and each training took approximately 2 h on an NVIDIA 3090 GPU with 24 GB of random-access memory.

### 2.6 Scale establishment

Considering that deep learning depends on hardware support, which limits clinical practice, we proposed a clinically practical scale for the rapid QST classification of CPs. We selected easily accessible features and analyzed the contribution and significance of each feature to construct a clinical scale based on multivariate logistic regression (22). The cutoff value of each feature was determined by the maximum AUC value.

### 2.7 Statistical methods

SPSS software (version 25.0) was used for univariate and multivariate logistic regression analyses and AUC value calculation. We use ITK-snap (University of Pennsylvania, www.itksnap.org) for the annotation of images, Python 3.7 for the processing of data, and PyTorch (version 1.7.1) for the construction of neural networks. Two-sided p-values<0.05 were considered significant.

## 3 Results

### 3.1 Patient characteristics

A total of 133 patients from the Affiliated Hospital of Southern Medical University were included in the study (**Table 1**). We diagnosed all patients according to operational findings of the tumor origin, including 29 (21.8%) patients with Q-CPs, 22 (16.5%) patients with S-CPs, and 82 (61.7%) patients with T-CPs. Patients with Q-CPs were younger (age 18.8 ± 16.3 years) and had smaller pituitary volumes (0.01 ± 0.01 cm^3^). Patients with T-CPs were more likely to present with hydrocephalus on images (59 in 82, 72%). Headache and visual impairment were the main symptoms among patients with three tumor types (63.2% and 69.2%, respectively). The enrolled patients showed a large tumor volume on MR images (21.9 ± 19.8 cm^3^).

**Table 1:** Characteristics of enrolled patients with QST classification.

Classification	ALL	Q	S	T
Number of patients	133	29	22	82
Male, (%)	77 (57.9)	14 (48.3)	11 (50.0)	52 (63.4)
Age, years	30.8 ± 20.4	18.8 ± 16.3	33.9 ± 17.2	34.2 ± 21.0
Course, months	38.6 ± 16.9	36.8 ± 13.3	34.6 ± 17.4	40.3 ± 17.9
Visual impairment, (%)	92 (69.2)	22 (75.9)	17 (77.3)	53 (64.6)
Headache, (%)	84 (63.2)	17 (58.6)	11 (50.0)	56 (68.3)
Hydrocephalus, (%)	81 (23.3)	14 (48.3)	8 (36.4)	59 (72.0)
Tumor volume, cm^3^	21.9 ± 19.8	24.2 ± 26.3	25.8 ± 26.2	20.0 ± 14.6
Pituitary volume, cm^3^	0.36 ± 0.30	0.01 ± 0.01	0.49 ± 0.30	0.45 ± 0.26

### 3.2 Automatic segmentation results

**Figure 4:**
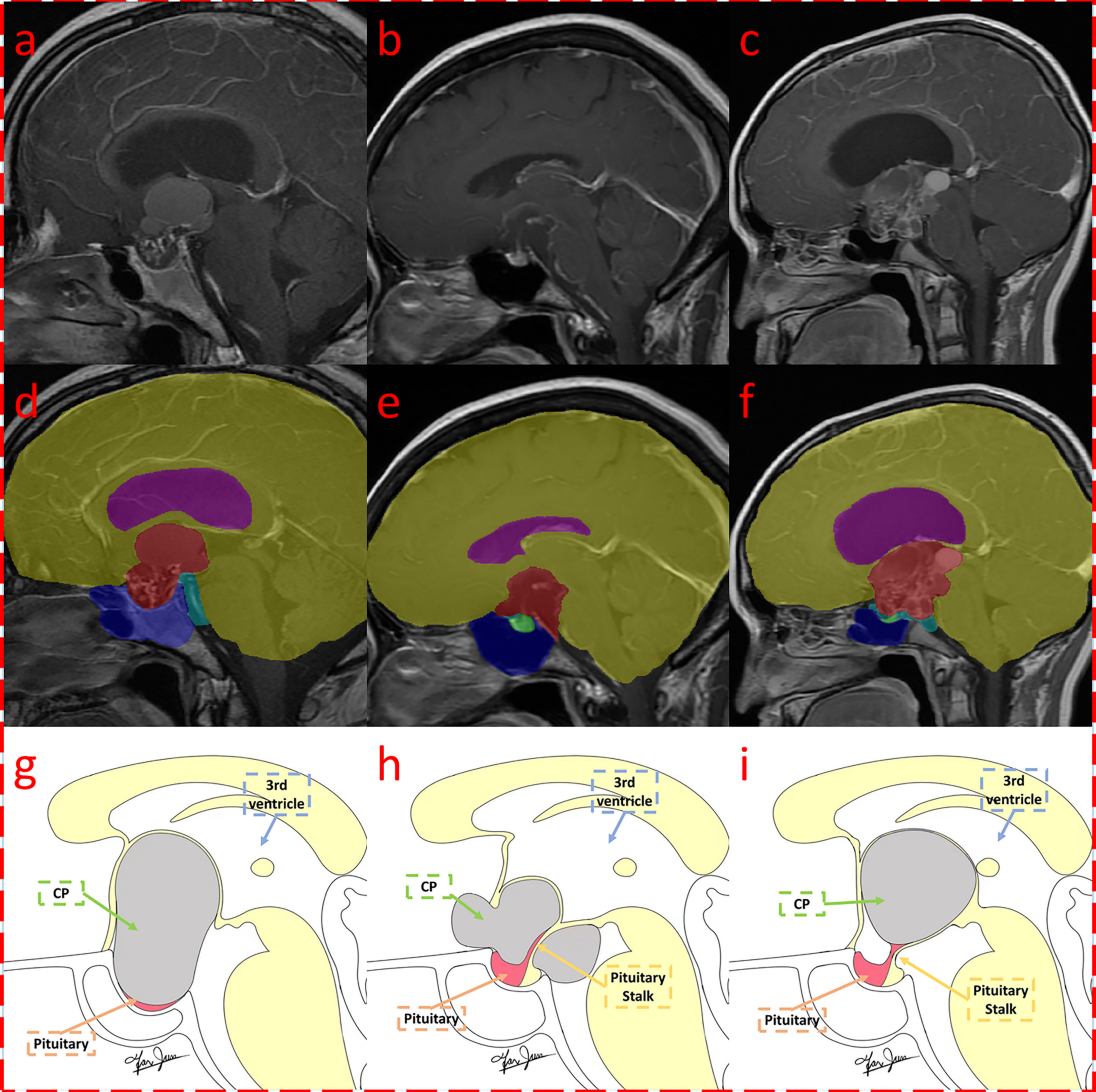
Segmentation result of craniopharyngioma with QST types. Three MRI with segmentations were shown, which were classified as Q **(A, D, G)**, S **(B, E, H)**, and T **(C, F, I)** subtypes according to the QST classification system. The first row **(A–C)** shows the original contrast-enhanced T1 image; the second row **(D–F)** shows the multitissue segmentations; and the third row **(G–I)** illustrates the QST classification system. The three columns are diagnosed as Q, S, and T types, respectively.

Due to the use of fivefold cross-validation, an average of 106 patients were selected for the training set, and 27 patients were selected for the testing set. The automatic segmentation achieved a Dice coefficient of 0.951 for tumors, 0.724 for pituitary, 0.877 for sphenoid sinuses, 0.974 for the brain, 0.737 for superior saddle cisterns, and 0.938 for superior saddle cisterns lateral ventricles. The average Dice coefficient of the six labeled classes was 0.8668. The high Dice coefficient of 0.951 for tumors indicates that our automatic segmentation model had precise CP recognition ability, which was the key for our subsequent work. The segmentation results are shown in **Figure 4**.

### 3.3 Classification model results

According to the segmentation results of deep learning and knowledge based on clinical experience, we automatically extracted a total of 34 clinical features as follows: the length of three diameters in three axes for six tissues (18 features), the volume of six tissues (six features), the position of the tumor relative to the brain in three axes (three features), the position of the tumor relative to the sellar region in three axes (three features), the relative location of the maximum transverse diameter of the tumor (one feature), the tumor volume in the sellar region (one feature), the tumor volume anterior to the sellar tubercle (one feature), and the aspect ratio of the tumor (one feature). By using deep learning extraction, we extracted 32 depth features; therefore, a total of 66 features were fused and input into the discriminator for learning. Similarly, a fivefold cross-validation was applied, with an average of 106 patients selected for the training set and 27 for the testing set. Finally, we achieved a classification accuracy of 0.9098 on average. **Table 2** shows the results of the automatic classification model, which showed high discriminatory ability for the Q and T types (sensitivity of 0.9656 and 0.9634 and specificity of 0.9423 and 0.8824, respectively) but the poor discriminatory ability for the S type (sensitivity of 0.6364, but specificity of 1.0).

**Table 2:** Performance comparison between deep classification network and clinical scale.

Deep classification network	Q	S	T	3type
Sensitivity	0.9656	0.6364	0.9634	NA
Specificity	0.9423	1	0.8824	NA
Youden index	0.9079	0.6364	0.8458	NA
Accuracy	0.9474	0.9398	0.9323	0.9098
Clinical scale	Q	S	T	3type
Sensitivity	0.9656	0.7273	0.8659	NA
Specificity	0.9519	0.9279	0.902	NA
Youden index	0.9175	0.6552	0.7679	NA
Accuracy	0.9549	0.8947	0.8797	0.8647

if the scores are equal, the priority of the diagnosis is T>S>Q.

The range of the score is 0–16 for three types and type with maximum score is diagnosed as the final type.

### 3.4 Clinical scale results

To further simplify the clinical process and improve the accuracy based on multivariate logistic regression, we selected nine characteristics to construct a clinical scale for ease of clinical measurement (**Table 3**). For each type of classification, the scores ranged from 0 to 16. Using this scale, the classification was made by choosing the class with the highest score among the three types. If the scores were the same, the T type was preferentially diagnosed, followed by the S type.

**Table 3:** Clinical scale for fast classification of craniopharyngioma with QST types.

Type	Q		S		T	
	component	points	component	points	component	points
Pituitary	not clearly seen	8	/		clearly seen	6
Tumor diameter (antero-posterior)	<3.5cm	3	/		/	
Tumor diameter (supero-inferior)	>3.5cm	2	/		/	
Ventricle dilatation	/		No	5	Yes	3
Position of tumor relative to the brain	Anterior	3	anterior	2	posterior	1
Location of the maximum transverse diameter	/		inferior	2	superior	2
Tumor volume in the pituitary fossa	/		/		<2.1cm^3^	4
Tumor volume front of the tuberculum sellae	/		>0.8cm^3^	3	/	
Aspect ratio	/		>1.1	4	/	

NA, not applicable.

According to the scale, we found that the inability to visualize the tumor was the most important characteristic for the diagnosis of Q-CPs, and at the same time, Q-CP tumors had larger supero-inferior diameters and smaller anteroposterior diameters, and their location was anterior. Important features for the diagnosis of S-CPs were having no significant ventricular dilatation, a large tumor volume anterior to the sellar tubercle, a relatively anterior location, a large aspect ratio, and the largest transverse diameter appearing inferiorly. For the T-CPs, the pituitary needed to be visible, the internal tumor volume in the saddle area was small, there was significant ventricular dilatation, the tumor location was relatively posterior, and the maximum transverse diameter appeared above.

As shown in **Table 2**, the diagnosis of the Q-CPs can reach a high accuracy of 0.9549, while the accuracy of the S- and T-CPs is low (0.8947 and 0.8797). Similar to the classification model, the sensitivity of the S-CPs was low according to this scale. Overall, the proposed scale achieved an accuracy of 0.8647 for the classification of three types of tumors.

## 4 Discussion

In this study, we proposed a notable multi-tissue segmentation standard that can display six adjacent morphological structures of CPs and established a multi-tissue automatic segmentation method for CPs, which achieved a segmentation Dice coefficient of 0.951 for tumors and an average Dice coefficient of 0.8526 for six tissues. Based on the results of automatic segmentation, we proposed an automatic classification model and a simple clinical scale for the QST classification, which achieved accuracies of 0.9098 and 0.8647, respectively.

### 4.1 Classifications and surgical outcomes of craniopharyngiomas

There are many kinds of CP classifications. Pascual classified intraventricular craniopharyngiomas (IVCs) into strict IVC and non-strict IVC and found that strict IVC had a worse prognosis (8). Samii classified CPs into grades I–V according to the relationship between the pituitary gland and adjacent structures and found that patients who underwent total resection had a worse neuroendocrine prognosis (9, 23). Kassam graded CPs according to their suprasellar extension (I–IV) and concluded that more skilled endoscopists were needed to expose the field of view and should also be familiar with anatomical knowledge for larger tumors (7). Cao reported that EEA should be considered the first choice for intrasuprasellar and suprasellar types in a four-type craniopharyngioma classification (24). Tang’s classification based on an endoscopic approach divided craniopharyngiomas into the central type and peripheral type and found that most of the central type had a poor stalk preservation rate, while hypothalamus damage was more common in the hypothalamic stalk type (one of the three subtypes of peripheral type) (11). Additionally, other classifications based on the anatomical structure of the sellar region show that the relationship between the tumor and these structures affects the surgical approach and prognosis of the tumor (25–27). The QST classification is based on the origin of the tumor, which can explain the process of tumor growth and provide guidance for surgery, such as stripping the site of origin. To some extent, it helps to select the surgical approach, such as EEA for Q type and TCA for T type, which will have a better prognosis of visual improvement and hypothalamic function (12).

### 4.2 The accuracy of automatic segmentation

Our proposed methods achieved a high Dice coefficient for CP segmentation, while segmentation methods for CPs have not been reported in previous studies. For one of the tumors in the sellar region, relevant literature showed that they can achieve a Dice coefficient of 0.940 for pituitary adenomas in CE-T1 and 0.742 for nasopharyngeal carcinoma in CT, reporting lower coefficient results than ours (16, 28). Second, the segmentation accuracies of some structures, such as the pituitary and suprasellar cistern, were low. The reasons could be listed as follows: first, the smaller the tissue volume, the more difficult it was to segment. As manual labeling errors were inevitable, the impact of errors on small tissues was more significant for the segmentation model than on large tissues. Second, these tissues were located near the tumor and were vulnerable to tumor compression. Even in some cases, we were not able to observe pituitary tissue or the pituitary stalk from the sagittal view, and the large deformation brought difficulties in the segmentation process. Previous studies have shown that automatic segmentation of the pituitary gland adjacent to pituitary tumors is also difficult, with a low Dice coefficient of 0.6, indicating that pituitary tissue is difficult to segment when there is tumor compression (16). Overall, we achieved a segmentation Dice coefficient of 0.951 for tumors and an average Dice coefficient of 0.8526 for all tissues, indicating that our model can perform accurate segmentation for most clinical CPs.

### 4.3 Comparison of the extracted features among QST types

We hoped to explain a tumor by its originating location and growth characteristics. The origin of tumors of the QST types can start from the middle lobe of the pituitary gland and proceed to the mantle segment of the pituitary stalk sleeve to the top of the pars tuberalis. The farther the distance between the pituitary gland and the tumor, the lighter the compression of the pituitary. The tumor compression that led to the pituitary of the Q type constituted the most difficult case to distinguish, having a large tumor volume in the pituitary fossa, whereas the pituitary of the T type was the easiest to distinguish, with a small tumor volume in the pituitary fossa. From the aspect of the growth direction, the pituitary fossa is surrounded by bony structures with less deformation, resulting in the Q-CPs pushing or breaking the saddle diaphragm and growing upward. Therefore, S-CPs have a smaller anteroposterior diameter and a larger supero-inferior diameter.

S-CPs originate from the arachnoid sleeve segment of the pituitary stalk and are surrounded by a suprasellar cistern with less pressure. It easily grows toward the suprasellar cistern with a transverse growth pattern, resulting in a large anteroposterior diameter and a positive pyramid-like structure. Therefore, S-CPs with an anterior growth pattern can be characterized by a large volume anterior to the pretuberculum sellae and are located anteriorly relative to the brain.

T-CPs originate from the top of the pars tuberalis and the loose segment of the arachnoid membrane, and they can easily invade the floor of the third ventricle. With a multidirectional growth pattern, T-CPs compress the lateral ventricle, resulting in ventricular dilatation. T-type tumors with an irregular growth pattern show an inverted pyramid-like structure, and they are located posteriorly relative to the brain (29).

### 4.3 Comparison of discriminatory abilities among different types

The sensitivity and specificity of the Q type were relatively high, while the sensitivity and Youden value of the S type were relatively small. Considering the origin position being a bottom–up order from Q type to T type, the S-CPs were in the middle position, resulting in the invasion of the pituitary fossa and compression of the ventricle, which was difficult to distinguish from S and T types. Because of its central location of origin, the tumor can undergo transverse growth, which is different from the Q and T types. Therefore, a full understanding of the growth pattern of S-CPs could be beneficial to increase diagnostic accuracy.

### 4.4 Limitations

The surgical records of many hospitals do not report the origin of the tumor, greatly limiting the enrolled population size of retrospective studies. At the same time, this also limits the ability to retrospectively carry out multicenter studies. Second, the included CPs were relatively large and caused significant compression on the surrounding tissues. Moreover, large T-type tumors originating from the top of the pars tuberalis can grow into the pituitary fossa, which brings great difficulties to classification. Third, many studies consider hypothalamus function as an important prognostic factor, but we did not segment the hypothalamus in the segmentation procedure, which brings difficulties in the future analysis of hypothalamus function. In future studies, we recommend documenting the location of the origin of CPs in the surgical records to facilitate the study of the QST classification at the clinic. At present, the total surgical resection rate of CPs varies greatly among different regions, while the postoperative mortality rate remains high in inexperienced hospitals. Future research that uses innovative molecular imaging methods may help us better understand the formation of tumors and how they interact with the hypothalamic–pituitary axis.

## 5 Conclusion

We proposed a multi-structure segmentation method for craniopharyngioma based on deep learning, which achieves a Dice coefficient of 0.951 for segmenting tumors and an average Dice coefficient of 0.8668 for six classes. The proposed segmentation method can be used not only for three-dimensional reconstruction but also for intraoperative navigation. Because the QST classification is important for both preoperative surgical planning and postoperative prediction, we also proposed a classification model that can automatically classify tumors into three subtypes based on the automatic segmentation method. This classification achieved an accuracy of 0.9098. The clinical classification scale that we proposed achieved an accuracy of 0.8647 using sagittal MRI. Considering that an increasing number of studies have shown that deep learning greatly improves clinical work efficiency, we suggest that the automatic segmentation and classification methods designed in this study could be used as the primary method for identifying craniopharyngiomas rather than subjective judgments based on human experience.

## Data availability statement

The raw data supporting the conclusions of this article will be made available by the authors, without undue reservation.

## Ethics statement

Ethical review and approval was not required for the study on human participants in accordance with the local legislation and institutional requirements. Written informed consent from the participants’ legal guardian/next of kin was not required to participate in this study in accordance with the national legislation and the institutional requirements. Written informed consent was obtained from the individual(s), and minor(s)’ legal guardian/next of kin, for the publication of any potentially identifiable images or data included in this article.

## Author contributions

XY, HW and CJ initiated the study. BL, WF and JFa enrolled patients. MF and YF performed segmentation. HW, SL, and YF established the workflow. HW, XY, and JFu wrote the draft. RW and SQ revised and modified the draft. All authors approved the final version of the manuscript.
